# The African coalition for research and communication on abortion: shifting power, building capacity, defending rights

**DOI:** 10.1080/26410397.2026.2622222

**Published:** 2026-02-05

**Authors:** Naa Dodoo, Akanni Akinyemi, Pierre Akilimali, Wina Sangala, Oladimeji Ogunoye, Beniel Agossou, Akinrinola Bankole, Delayehu Bekele, Georges Guiella, Jewelle Methazia, Ritah Mukashyaka, Margarate Munakampe, Onikepe Owolabi, Susheela Singh

**Affiliations:** a Director, Human Capital Development, African Institute for Development Policy, Lilongwe, Malawi; b Professor of Demography and Social Statistics, Department of Demography and Social Statistics, Obafemi Awolowo University, Ile-Ife, Nigeria; c Professor, Director of Patrick Kayembe Research Center and Head of the Department of Research at the National Public Health Institute, Kinshasa, Democratic Republic of Congo (DRC); d Consultant Facilitator, ACORCA-COARCA, Lilongwe, Malawi.; e Consultant Facilitator, ACORCA-COARCA, Ile-Ife, Nigeria; f Technical Adviser in Health Systems, Centre ODAS, Abidjan, Côte d’Ivoire; g Senior Fellow, Guttmacher Institute, New York, NY, USA; h Professor of Obstetrics and Gynaecology, St. Paul Institute for Reproductive Health and Rights, Addis Ababa, Ethiopia; i Professor of Demography, Institut Supérieur des Sciences de la Population, Joseph Ki-Zerbo University, Ouagadougou, Burkina Faso; j Associate Project Director, Ibis Reproductive Health, Johannesburg, South Africa; k Research Associate, Health Development Initiative, Kigali, Rwanda; l Lecturer and Researcher, University of Zambia, Women in Global Health Zambia, Lusaka, Zambia; m Vice-President for International Research, Guttmacher Institute, New York, NY, USA; n Scholar Emerita, Guttmacher Institute, New York, NY, USA

**Keywords:** abortion, reproductive health, coalition, research capacity strengthening, Africa

## Background

Unsafe abortion remains an important, yet preventable, cause of maternal mortality and morbidity in Africa.^1^ Those already marginalised by poverty, geography, age or other forms of social exclusion bear this burden most acutely.^2,3^ A range of barriers limiting access to safe and comprehensive abortion care continues to exist throughout the entire African region. These include restrictive, complex or conflicting laws, policies and regulations surrounding abortion services and products; limited provider and public knowledge of the policy and legal environment and/or safe points of care; insufficient coverage and/or poor quality of services and products for both comprehensive abortion care and post-abortion care; and pervasive and persistent abortion stigma, inhibiting communication on abortion and the provision of care.^4–9^

Addressing these substantive challenges requires multi-pronged, well-resourced and strategic action. Central to this is the availability of good data and high-quality, policy-relevant evidence. While a small but important body of research on abortion in Africa exists – including a small number of population-representative abortion incidence and safety studies (many now dated), and a growing number of evaluative, clinical or social studies – the evidence base remains limited.^10^

Conducting and promoting abortion research can be challenging for a range of reasons, including stigma, legal restrictions, policy ambiguity and resultant fears of criminalisation. This is compounded by low prioritisation of abortion and wider reproductive health research, diminishing scientific funding for public health, as well as limited demand for evidence from policy-makers and/or legislators. These factors may discourage early-career researchers in the region from focusing on abortion. They can also find it challenging to openly share findings or have their data and insights accessed and acted upon. This situation is further exacerbated by the growing and alarming pushback on sexual and reproductive rights, choice and bodily autonomy occurring globally.

Consequently, there is a lack of publicly available data and research on abortion throughout the African region, resulting in underestimates of abortion incidence and severity.^11^ More specialised tools and methodologies are needed, as well as adequate resourcing for nationally representative surveys – costly yet crucial for high-level policy decision-making.

These factors have shaped the abortion research ecosystem on the continent, resulting in studies on abortion being primarily designed and led by institutions from outside the region. This international leadership may include decisions on the nature and locus of the study, grant management (and linked research agenda-setting), the technical and academic leadership on the study and/or first authorship. There is, though, a positive shift unfolding, with notable abortion research capacity strengthening efforts leading to a growing number of trained researchers in the region.^12^ This positive trajectory must continue so that research agendas on issues pertinent to Africa are set and led by scientists and research users from the region.

## An introduction to ACORCA-COARCA

The African Coalition for Research and Communication on Abortion – Coalition Africaine pour la Recherche et la Communication sur l’Avortement (ACORCA-COARCA) was created as a direct response to these intersecting needs and challenges.† Our bilingual (English-French) coalition represents more than a new organisational entity; it is a deliberate attempt to rethink how abortion research is produced, owned, and applied in Africa. At its centre lies a required shift in agenda-setting, governance, research skills and capacities and funding from Northern to African institutions. This transfer of power is both practical and symbolic: African institutions will set research priorities, directly manage funds, and lead studies directly, ensuring the data and insights address urgent questions on national, regional, continental, and global scales.

From the start, ACORCA-COARCA has been envisioned as a permanent feature on Africa's public health research landscape – a network capable of producing high-quality science and supporting member organisations to produce research while also leveraging its evidence base and expert membership to help defend reproductive rights in the face of ideological attacks. Central to this vision is shared learning and collaboration across the African region, as well as with partners outside of Africa – both as a support mechanism to address methodological or political challenges and to ensure the sharing of information on relevant research and advocacy initiatives. The coalition has also ensured the early and deep involvement of those who will use the research: advocates and health providers are among our current members, and our coalition is developing strategies and mechanisms to involve policy-makers, journalists and community activists. Their involvement is essential to ensure that research is not only scientifically rigorous, but also timely, relevant and actionable.

Equity and innovation are also defining features of the coalition. ACORCA-COARCA is committed to advancing equity in reproductive health by prioritising support for research on disparities in abortion access across Africa. The coalition members will work to identify and evaluate service delivery models, technologies and interventions that can improve access to quality care, especially for underserved regions and populations. Furthermore, by integrating both digital tools and a range of knowledge systems across the continent, ACORCA-COARCA also aims to develop and share contextualised alternative tools and methodologies to demonstrate the need or to identify cost-effective and sustainable solutions for scaling safe abortion services. This evidence will be used not only to inform policy but also to hold governments and healthcare providers accountable for meeting the needs of all populations.

### The coalition's origins and approach

ACORCA-COARCA emerged from open conversations among African sexual and reproductive health and rights (SRHR) researchers, practitioners, and allies. In 2021, the Guttmacher Institute, a global leader in the production and communication of research on abortion and wider SRHR, initiated a multi-country initiative focused on building sustainable abortion research and advocacy expertise, *Strengthening Capacity and Catalysing Change for Research and Communications Partners in Low and Middle-income Countries.* The work resulted in calls by the programme's African partners and Guttmacher staff to support an Africa-based and -led research and communication entity.

Through early engagement with African research partners, a networked coalition structure was determined to be an optimal organisational structure that could build on the expertise and unique capacities of its diverse members. Coalitions are proven vehicles for amplifying issues, pooling resources and expertise, increasing visibility and credibility, and uniting diverse voices for a common purpose,^13,14^ especially vital when abortion researchers can face ideological attacks from the media or policy-makers. A supra-organisational structure also ensures that the coalition does not become a competitor to its member organisations, but rather works to support them with their own development and effectiveness. Through collective action, members can pair complementary skills, communicate findings more effectively, and reach decision-makers via varied relationships and networks. Greater visibility and media attention are more likely, raising awareness and building consensus for change. Political influence grows with the coalition's breadth and diversity, which is essential in today's increasingly restrictive climate.

ACORCA-COARCA will actively seek out, join and work alongside other effective advocacy and research networks seeking to improve access to comprehensive abortion care and to strengthen research capacities in Africa, including: the MAMA Network (Mobilising Activists around Medical Abortion)‡; the recently launched Catalysts network§ and the Africa Research Excellence Fund (AREF).**

ACORCA's co-creation workshop in 2024 brought together experienced and emerging researchers, institutional leaders, civil society and technical experts from across linguistic and geographic boundaries. Participants became the coalition's “founding members”, representing 16 institutions from Central, East, Southern and West Africa. Over several days, participants discussed challenges to research production, communication and uptake, as well as evidence-based strategies to build capacities and build effective coalitions. Using these inputs, the group developed the building blocks for its future work: a shared vision and mission; aims and strategies to achieve them; working principles and values; and a workable governance structure ensuring participation, representation and accountability.

### Early activities and structure

In its initial months and with some seed funding from the Guttmacher Institute, ACORCA-COARCA has begun translating its vision into reality. ACORCA's theory of change centres around three core aims, from which flow associated activity plans:

1. **To support and empower abortion researchers**, through: fundraising support; knowledge exchange; support to young, female and under-represented researchers; ethics board training; strategic partnerships; and research prioritisation.
2. **To strengthen abortion research capacities**, through: research training and mentorship; small grants and fellowships; development of multi-country research partnerships; and technical support to non-governmental and community-based organisations.
3. **To communicate and advocate with evidence**, through: training and mentorship on research communication and public engagement; stakeholder mapping and engagement; media and public engagement activities; and opposition monitoring.

We have elected an 11-member Executive Committee, representing organisations from Central, East, Southern and West Africa, led by three co-chairs (two male, one female; two anglophone, one francophone); have established a Secretariat office, hosted in Rwanda by one of our Founding Members, Health Development Initiative (HDI); and a small Advisory Council of abortion research, advocacy and programming experts will provide technical and strategic guidance, help identify new partnerships, networks and opportunities and increase visibility. One initial activity has been the implementation of a small grants programme to strengthen the abortion research capacity of early-career researchers from six countries and connect them with senior African abortion researchers from ACORCA's Executive Committee, encouraging reciprocal skills transfer and mentorship.

The coalition officially launched at the International Conference on Family Planning (ICFP) in November 2025 and is now seeking to expand its membership base, welcoming other research institutions and partners into its network, including individuals and institutions from outside of Africa as observer members.

## Looking ahead

ACORCA-COARCA's vision is ambitious but grounded in sustainability. The continued development of the network will require ongoing partner engagement, in particular with researchers and advocates based in North Africa and in Lusophone Africa who have been absent to date. Plans also include the establishment of sub-regional partnership fora to strengthen sub-regional collective action. The coalition is keen on raising funds both for its own research activities and to support its members. It aims to facilitate multi-country studies led entirely from Africa, focusing on members’ priorities in their unique contexts while pooling resources, tools, and engagement approaches.

Capacity strengthening continues to be needed and will include efforts to support the exchange of technical expertise on complex abortion measurement studies from institutions with significant expertise on these topics with African epidemiologists and demographers, as well as broader training on abortion-specific research methods and the effective communication of findings. We will make a deliberate commitment to elevate female African researchers, recognising gender equity in leadership as central to whose knowledge is valued. Efforts will be made to involve research users and engage the media, health providers and policy-makers through sub-grants to members.

For funders and partners, ACORCA-COARCA is more than an opportunity to support single projects; it is a chance for real structural change. Funders need to acknowledge and recognise the strength and capacity of African institutions to conduct rigorous reproductive health research and provide direct and sustained funding to enable them to do so. By consolidating African leadership and user engagement, and maintaining commitments to innovation and equity, the coalition addresses long-standing gaps in the abortion research ecosystem. A blended financing approach – programmatic donor grants, scientific research grants and affordable membership fees – will ensure resilience and independence. The dividends will be seen in stronger health systems, better policies, improved protection of reproductive rights, and a new generation of African abortion researchers whose authority is recognised continent-wide and beyond. With lives and rights at stake, supporting and consolidating this shift is more urgent than ever.

## Footnotes

† The founding members of ACORCA are: The African Institute for Development Policy (AFIDEP), Malawi; The Academy for Health Development (AHEAD), Nigeria; The African Population and Health Research Center (APHRC), Kenya; Centre ODAS (Organisation pour le dialogue pour l'avortement Sécurisé), Côte d'Ivoire; le Centre de recherche en reproduction humaine et en démographie (CERRHUD), Benin; Centre for Research, Evaluation Resources, and Development (CRERD), Nigeria; Chama cha Uzazi na Malezi Bora Tanzania (UMATI), Tanzania; Health Development Initiative (HDI), Rwanda; Ibis Reproductive Health, South Africa; Ipas, Ethiopia; Institut Supérieur des Sciences de la Population (ISSP), Burkina Faso; Population Council, Kenya; St. Paul Institute for Reproductive Health and Rights (SPIRHR), Ethiopia; University of Kinshasa/School of Public Health/Patrick Kayembe Research Center, Democratic Republic of Congo; University of Ghana/Regional Institute for Population Studies, Ghana (RIPS); University of Zambia/School of Public Health, Zambia.

‡ An African movement of activists working together to share evidence-based and stigma-free information about self-managed medical abortion (https://mamanetwork.org/).

§ An alliance of non-governmental organisations (NGOs) committed to championing quality abortion care across the continent (https://catalystsafrica.org/).

** Founded in 2015 to improve the quality and impact of health research in Africa (https://africaresearchexcellencefund.org.uk/).

## Provenance statement

This article was not commissioned and went through internal review.
